# A Mendelian randomization analysis reveals the multifaceted role of the skin microbiota in liver cancer

**DOI:** 10.3389/fmicb.2024.1422132

**Published:** 2024-07-24

**Authors:** Xiaoxue Wang, Zexin Zhu

**Affiliations:** ^1^Department of Dermatology, The Second Affiliated Hospital of Xi'an Jiaotong University, Xi'an, China; ^2^Department of Surgical Oncology, The Comprehensive Breast Care Center, The Second Affiliated Hospital of Xi'an Jiaotong University, Xi'an, China

**Keywords:** causal impact, European descent, liver cancer, Mendelian randomization, skin microbiota

## Abstract

**Background:**

Hepatocellular carcinoma (HCC, or hepatic cancer, HC) and cholangiocarcinoma (CCA, or hepatic bile duct cancer, HBDC) are two major types of primary liver cancer (PLC). Previous studies have suggested that microbiota can either act as risk factors or preventive factors in PLC. However, no study has reported the relationship between skin microbiota and PLC. Therefore, we conducted a two-sample Mendelian randomization (MR) study to assess the causality between skin microbiota and PLC.

**Methods:**

Data from the genome-wide association study (GWAS) on skin microbiota were collected. The GWAS summary data of GCST90018803 (HBDC) and GCST90018858 (HC) were utilized in the discovery and verification phases, respectively. The inverse variance weighted (IVW) method was utilized as the principal method in our MR study. The MR-Egger intercept test, Cochran's *Q*-test, MR-Pleiotropy RESidual Sum and Outlier (MR-PRESSO), and leave-one-out analysis were conducted to identify the heterogeneity and pleiotropy.

**Results:**

The results showed that *Veillonella (unc.)* plays a protective role in HBDC, while the family Neisseriaceae has a positive association with HBDC risk. The class Betaproteobacteria, *Veillonella (unc.)*, and the phylum Bacillota (Firmicutes) play a protective role in HC. *Staphylococcus epidermidis, Corynebacterium (unc.)*, the family Neisseriaceae, and *Pasteurellaceae* sp. were associated with an increased risk of HC.

**Conclusion:**

This study provided new evidence regarding the association between skin microbiota and PLC, suggesting that skin microbiota plays a role in PLC progression. Skin microbiota could be a novel and effective way for PLC diagnosis and treatment.

## Introduction

Hepatocellular carcinoma (HCC, or hepatic cancer, HC) and cholangiocarcinoma (CCA, or hepatic bile duct cancer, HBDC) are the two major types of primary liver cancer (PLC). It can be noted statistically that the incidence of PLC (both HC and HBDC) has continued to increase over the past several years (Rahib et al., 2014; Petrick et al., 2016). The diagnosis and treatment strategies for HC and HBDC are, in some respects, similar based on their baseline clinical features. The diagnosis of PLC is primarily based on radiologic, serologic, and/or pathologic methods (Chaisaingmongkol et al., 2017). Over the past few years, a growing number of therapeutic interventions have been approved for the treatment of PLC, but most of them only provide limited survival benefits. For example, resection or transplantation is a conventional treatment of choice for PLC; however, surgical management is challenging because of the complex hepatobiliary anatomy and chronic liver damage (Gunasekaran et al., 2021; Sapisochin et al., 2022). Chemotherapy or treatment with multikinase inhibitors (for example, sorafenib and lenvatinib for HC or dabrafenib and trametinib for HBDC) provides only a minor prolongation of overall survival and a marginal increase in quality of life (Subbiah et al., 2020; Li et al., 2021). Although research on the genetic landscape of PLC has grown substantially over the past few years, various questions remain unexplored (Li et al., 2021). Given the complex histology and biology of PLC, it is essential to explore new biomarkers and potential intervention measures to delay the progression of PLC.

The microbiome affects the function of several organs in the body. Increasing research has highlighted the complex link between the microbiome and a number of human diseases (Gopalakrishnan et al., 2018). Accordingly, the microbiome plays an important role in a host of metabolic disorders, such as diabetes, obesity, hypertension, and non-alcoholic fatty liver disease (NAFLD; Michels et al., 2022). Recently, emerging studies have indicated that the microbiome is closely related to different types of cancer. Tumor-coating enterotoxigenic *Bacteroides fragilis* recruits other bacteria and immune cells to the tumor site and boosts colorectal cancer progression (Dejea et al., 2018). *Malassezia globose* contributes to tumorigenesis, tumor growth, and gemcitabine resistance in pancreatic ductal adenocarcinoma (Aykut et al., 2019). *Fusobacterium nucleatum* is related to tumor infiltration of Treg lymphocytes in a chemokine-dependent fashion, promoting aggressive tumor behaviors (Yamamura et al., 2016).

Mendelian randomization (MR) utilizes one or more genetic variants as instrumental variables (IVs) based on genome-wide association studies (GWAS). MR studies can infer the causal effects of exposure on an outcome. Several studies based on MR analysis showed a causal association between gut microbiota and illnesses. For instance, recent findings have shown a significant association between gut microbiota and diabetes, celiac disease (Xu et al., 2022), and various types of cancer (Long et al., 2023). Generally, the gut is the primary habitat of human microbiota; meanwhile, thriving microbial populations exist throughout most of the body, including the skin, genital tracts, and oral and respiratory organs (The Human Microbiome Project Consortium, 2012). Unlike gut microbiota, the role of the skin microbiota in cancer progression is largely unknown. To the best of our knowledge, no study has yet investigated the causal effect of the skin microbiota on PLC risk using MR. Our investigation aimed to utilize MR to explore the role of risk variants of the skin microbiota as IVs for PLC.

## Materials and methods

### Study design

**Figure 1:**
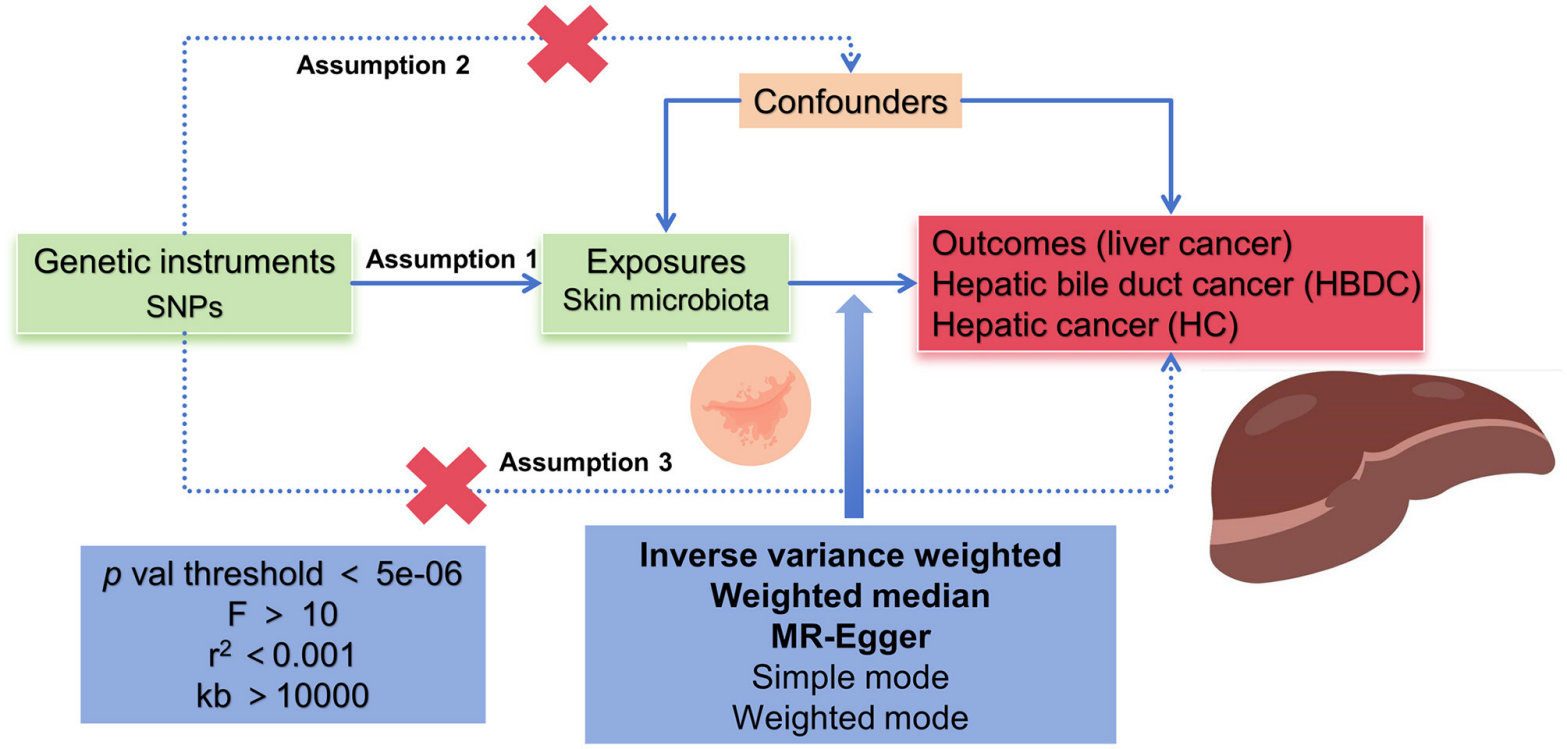
Flowchart schematic diagram illustrating the principles of MR analysis in this study.

According to the MR framework (Figure 1), three key assumptions were included in the study. (1) Relevance assumption: single nucleotide polymorphisms (SNPs) that were substantially linked to exposures were used as IVs. (2) Independence assumption: these SNPs (IVs) did not show any correlation with the relevant confounding factors. (3) Exclusivity assumption: These SNPs (IVs) affect outcomes only through their effects on exposure (Boef et al., 2015; Bowden et al., 2017).

### Data sources

The skin microbiota GWAS data were obtained from two German cohorts, KORA FF4 (*n* = 324, Holle et al., 2005) and PopGen (*n* = 273, Nöthlings and Krawczak, 2012), as summarized by Moitinho-Silva et al. (2022). Participants for the KORA FF4 cohort were selected from the youngest age group ranging from 39 to 48 years. They were previously genotyped as part of the KORA S4 Survey and were recruited from the southern German city of Augsburg and its two surrounding counties (Holle et al., 2005; Moitinho-Silva et al., 2022). PopGen cohort participants were randomly recruited via the local population registry in Kiel, Germany, and from blood donors of the University Hospital Schleswig-Holstein, Campus Kiel (Nöthlings and Krawczak, 2012; Moitinho-Silva et al., 2022). More detailed descriptions can be found in the source publication. A total of 1,656 skin samples were analyzed. The skin samples were taken from dry, moist, sebaceous, and forehead skin microenvironments. High-throughput gene sequencing technologies were used to analyze the skin microbiota of a large cohort of individuals; microbial community profiles were obtained from the sequencing of the V1-V2 regions in the 16S ribosomal RNA (rRNA) gene. Genome-wide association analyses were conducted on the univariate relative abundances of individual bacteria (amplicon sequence variants; ASVs) and non-redundant taxonomic groups ranging from genus to phylum levels. The GWAS data related to PLC were obtained from the IEU OpenGWAS project, based on research by Sakaue et al. (2021), with GWAS ID: ebi-a-GCST90018803 for HBDC and ebi-a-GCST90018858 for HC. The dataset included 476,091 individuals, comprising 832 cases and 475,259 controls, and encompassed 24,196,592 SNPs. The study population consisted of individuals of European descent. All participants provided informed written consent, and all studies were reviewed and approved by the institutional ethics review committees of the institutions involved.

### The selection of instrumental variables

Related IVs for the MR analysis followed particular principles. SNPs should be associated with exposures at the locus-wide significance level, *p* < 5e-06. In addition, the linkage disequilibrium (LD) coefficient *r*^2^ should be <0.001 and not closely related (clumping window more than 10,000 kb) to ensure the independence of exposure instruments. We used the *F*-statistics to measure the strength of the IVs, the values of which were more than 10 (Bowden et al., 2015).

### MR analysis

Causal associations between skin microbiota and PLC were determined by conducting MR and reverse causality analyses. In the exposure-outcome analysis, we employed MR with more than two SNPs serving as IVs. In our MR analysis, five methods, namely, the inverse variance weighted (IVW) method, the weighted median method, the MR-Egger method, the simple mode, and the weighted mode, were utilized. The IVW method was used as the primary statistical analysis method in our MR analysis for evaluating causal effects (Boef et al., 2015; Bowden et al., 2015, 2017).

The heterogeneity of the chosen SNPs was evaluated by conducting Cochran's *Q*-test, where a *p*-value of more than 0.05 suggested a lack of heterogeneity. The random effects model was used once significant heterogeneity had been identified. We evaluated the possible bias from horizontal pleiotropy using the weighted median and MR-Egger regression models in order to gauge the robustness of the IVW method. The MR-Pleiotropy RESidual Sum and Outlier (MR-PRESSO) test was conducted to assess outliers that might have been influenced by horizontal pleiotropy. The causal effect estimates for individual variants were displayed using a scatter plot. Thereafter, we performed a leave-one-out analysis to examine the stability of the results in the context of a single SNP's influence and presented the findings in a forest plot (Boef et al., 2015; Bowden et al., 2015, 2017).

All statistical analyses were conducted using R software (Version 4.3.2) with the TwoSampleMR package (Version 0.5.8). The statistical significance level was set at a *p-*value of <0.05. Pooled odds ratios (ORs) with a 95% confidence interval (CI) were calculated.

## Results

### MR analysis

**Figure 2:**
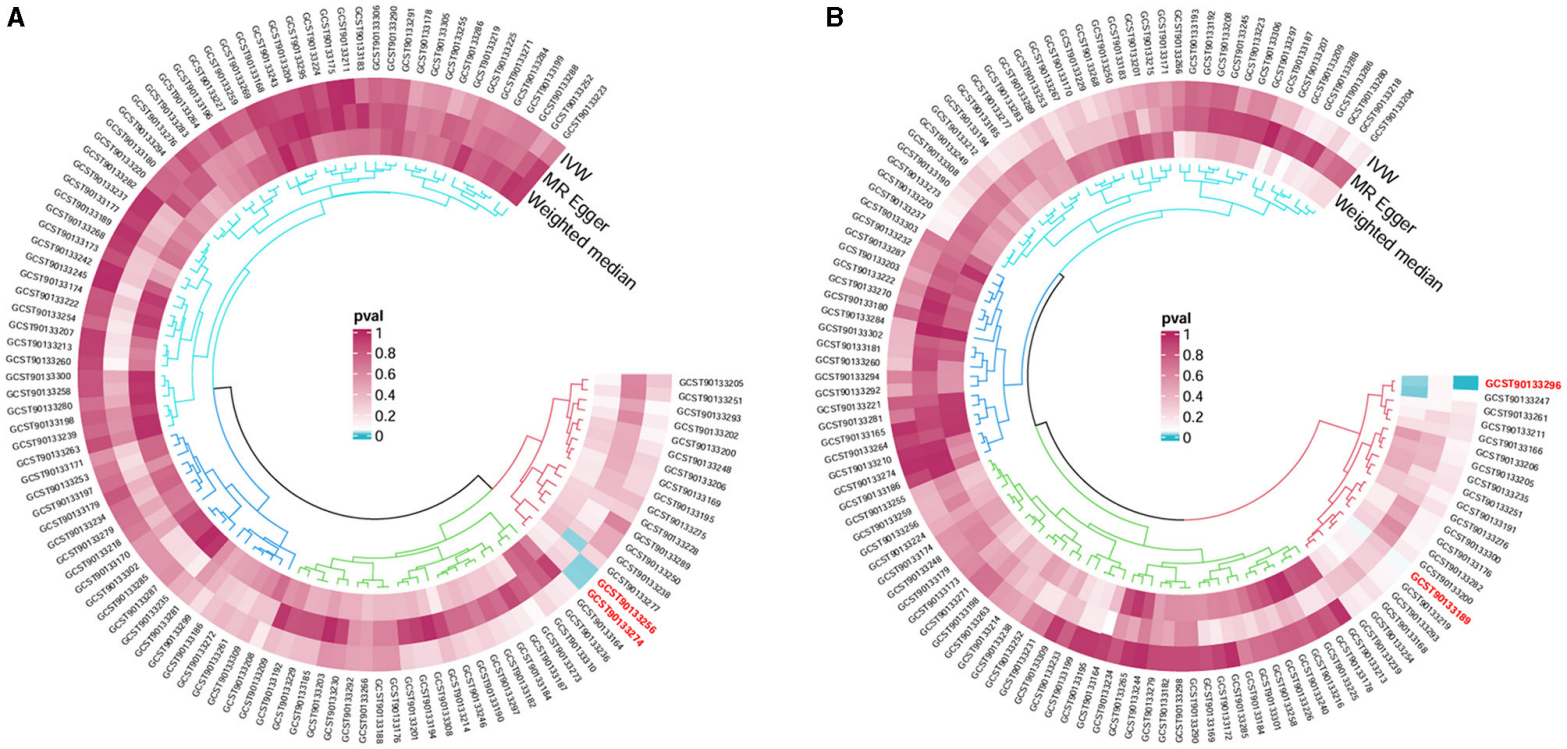
Skin microbiota and their causal association with HBDC. **(A)** KORA FFA cohort; **(B)** PopGen cohort.

**Figure 3:**
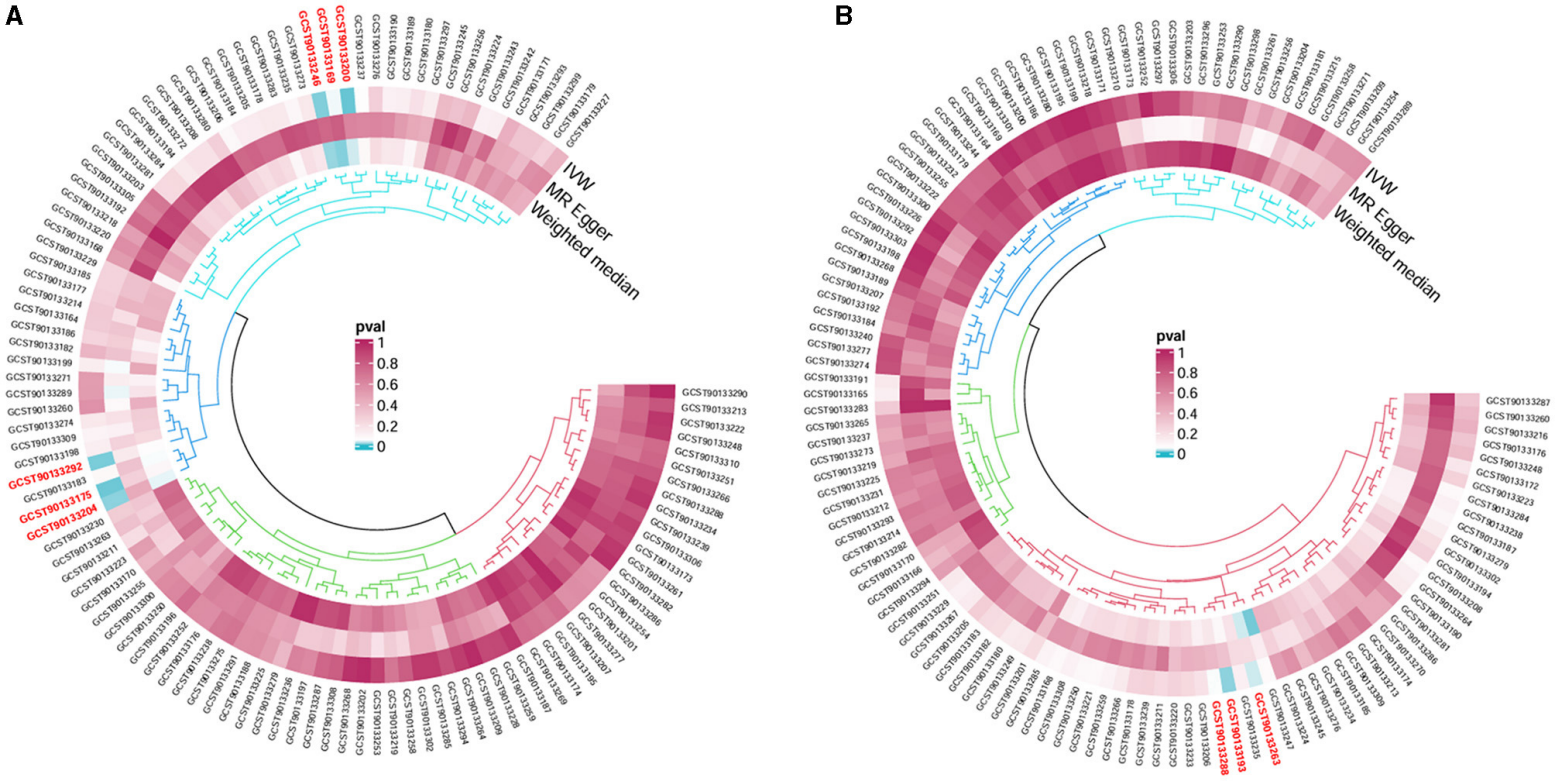
Skin microbiota and their causal association with HC. **(A)** KORA FFA cohort; **(B)** PopGen cohort.

**Figure 4:**
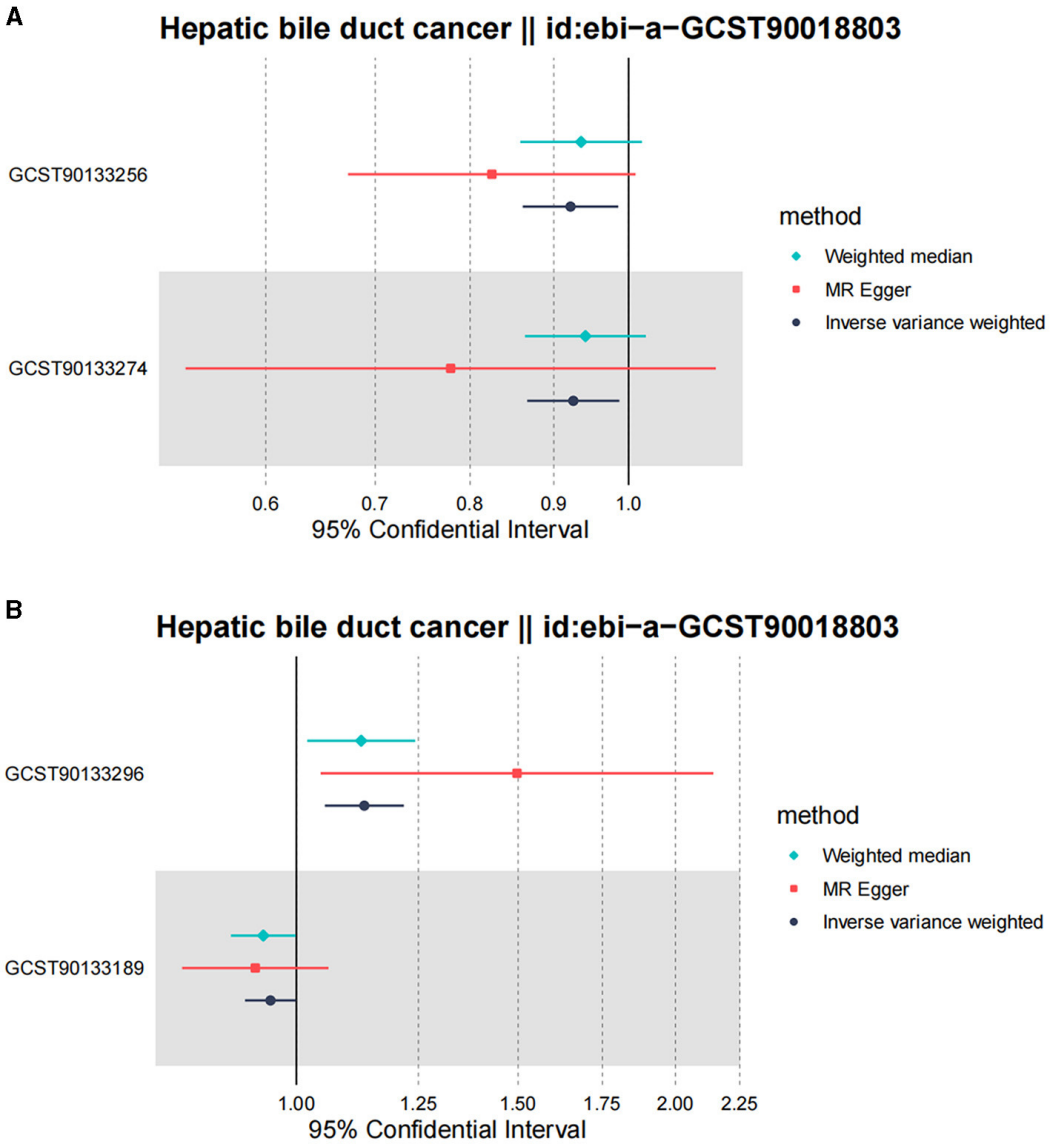
Forest plot of Mendelian randomization analysis for skin microbiota and their causal association with HBDC. **(A)** KORA FFA cohort, Id for exposure, GCST90133256: ASV011 unknown; GCST90133274: ASV070 [*Veillonella (unc.)*]; **(B)** PopGen cohort, Id for exposure, GCST90133296: the family: Neisseriaceae*;* GCST90133189: ASV011 unknown.

**Figure 5:**
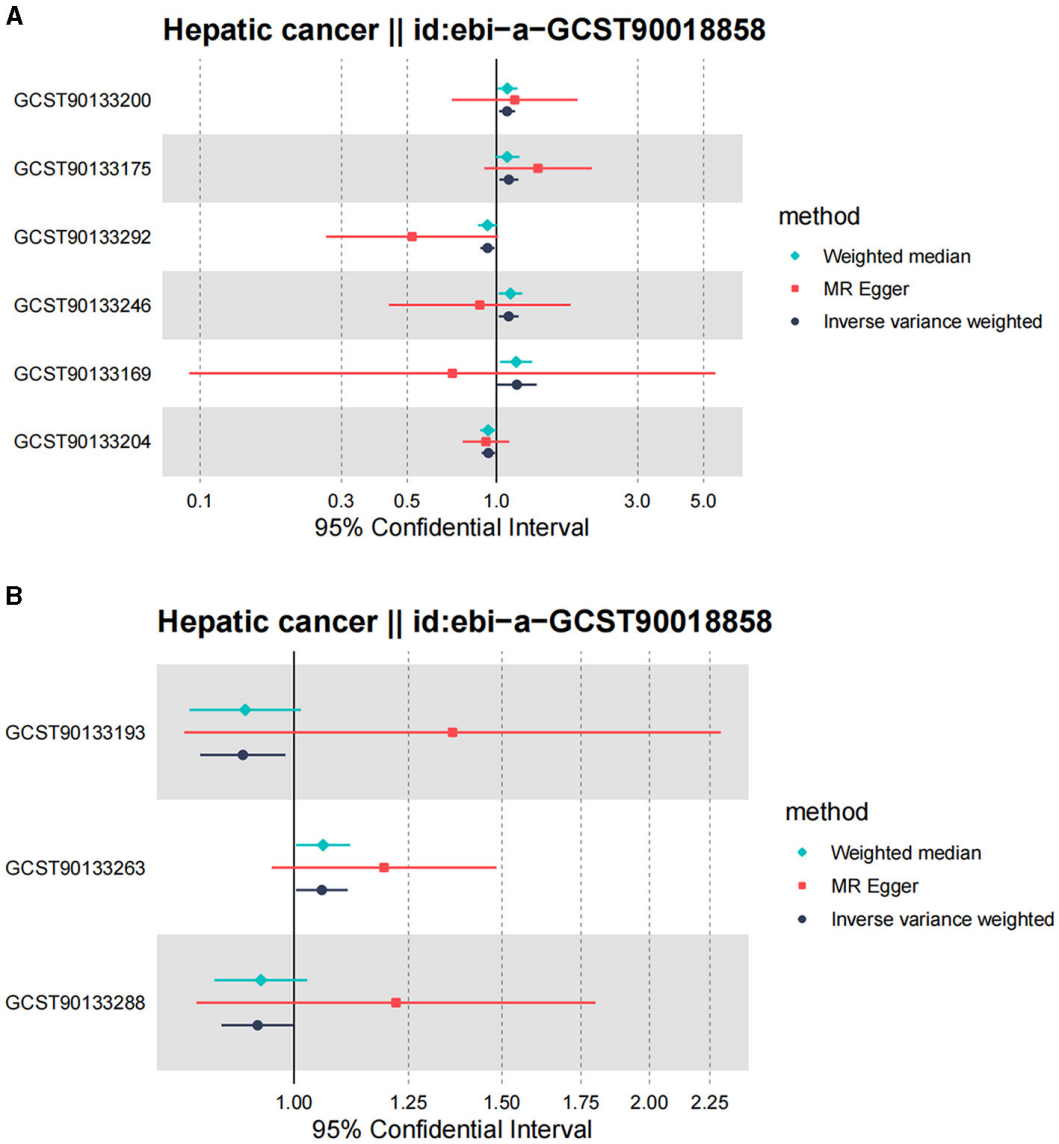
Forest plot of Mendelian randomization analysis for skin microbiota and their causal association with HC. **(A)** KORA FFA cohort: Id for exposure, GCST90133169: ASV013 [*S. epidermidis*]; GCST90133175: ASV004 [*Corynebacterium (unc.)*]; GCST90133200: the family: Neisseriaceae; GCST90133204: ASV005 unknown; GCST90133246: ASV019 [*Pasteurellaceae sp*.]; GCST90133292: ASV070 [*Veillonella (unc.)*]. **(B)** PopGen cohort. Id for exposure, GCST90133193: the phylum: Bacillota (Firmicutes); GCST90133263: ASV039 unknown; GCST90133288: the class: Betaproteobacteria.

**Figure 6:**
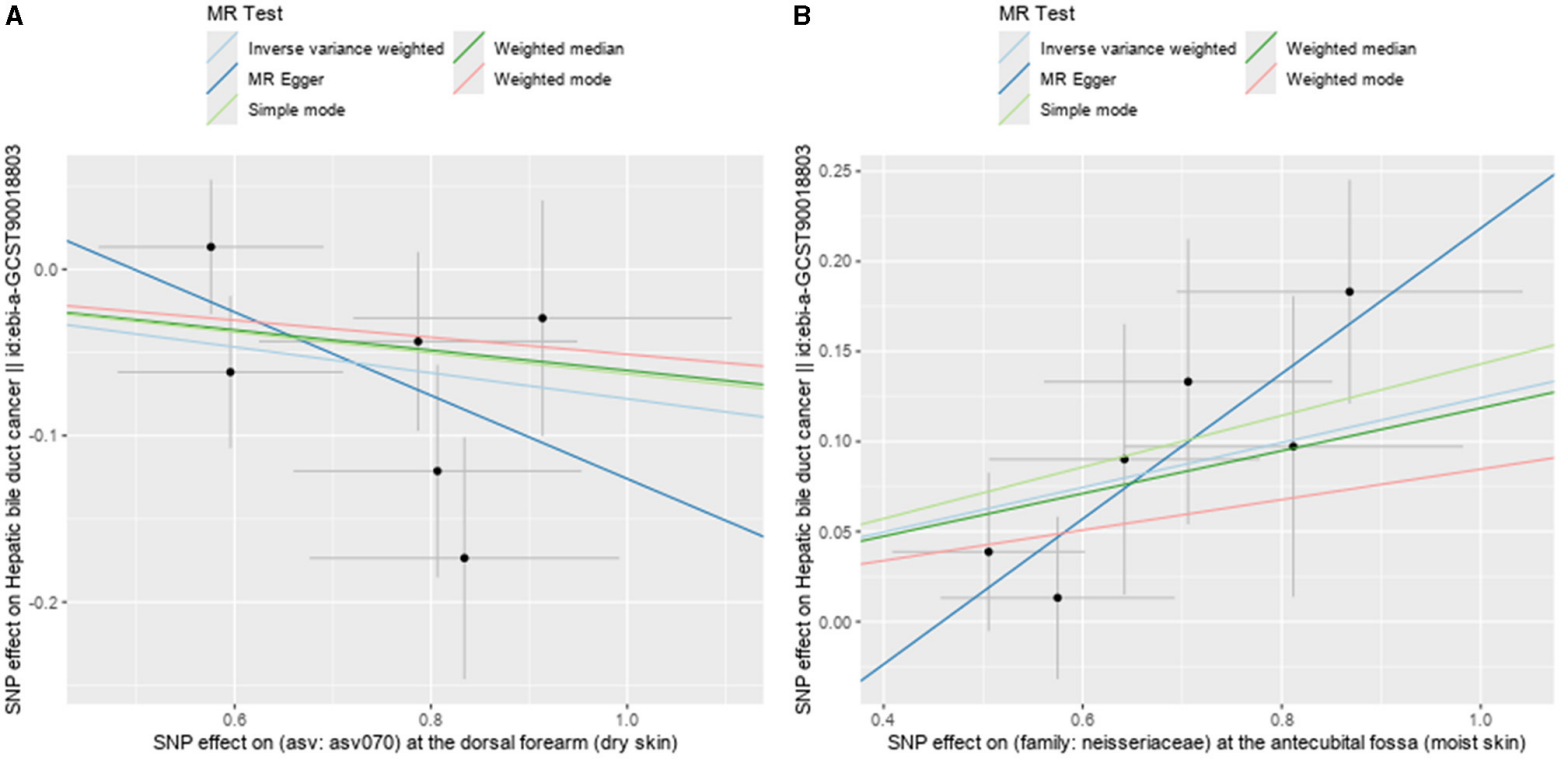
Scatter plots showing significant causal effects between skin microbiota and HBDC. **(A)** ASV070 [*Veillonella (unc.)*]; **(B)** the family: Neisseriaceae.

**Figure 7:**
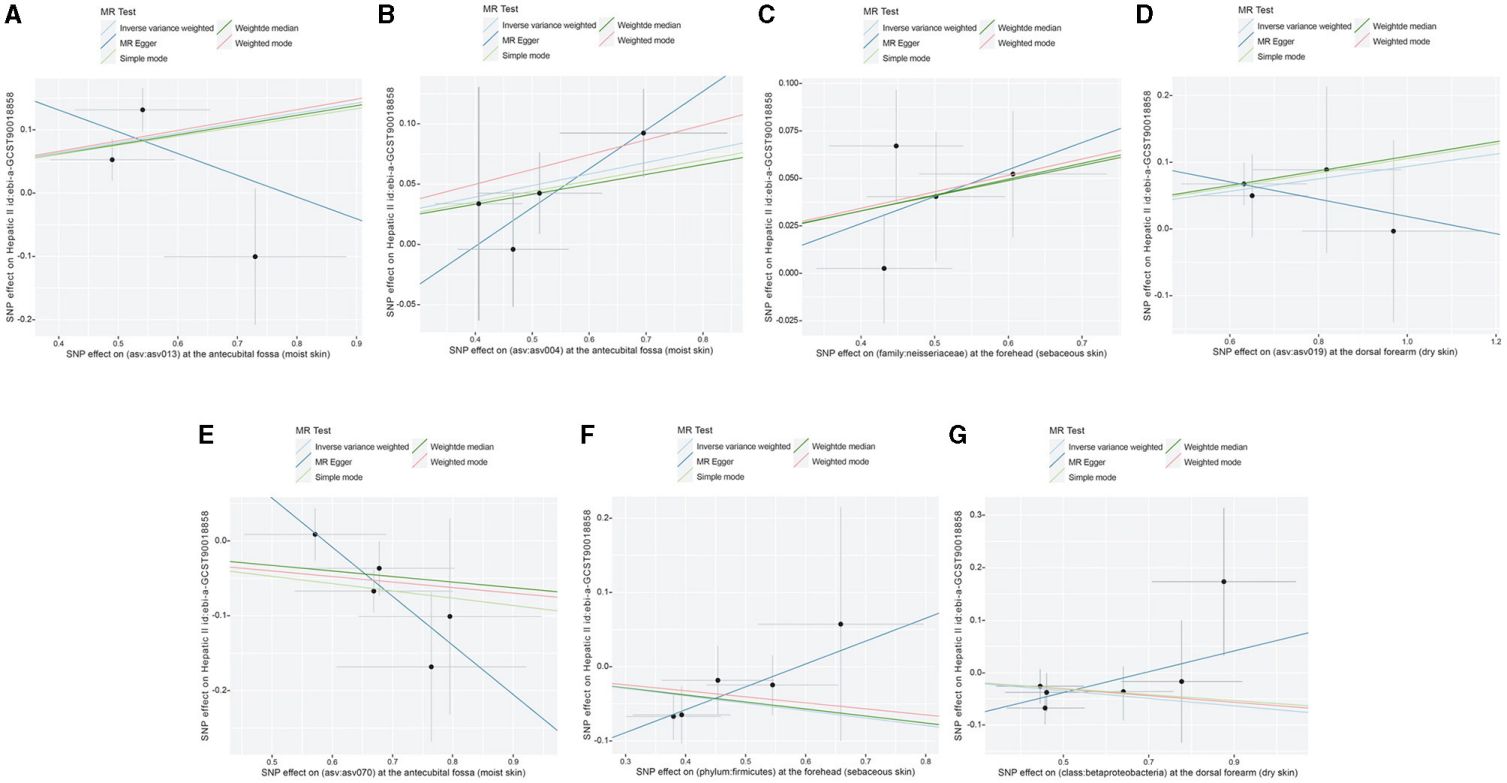
Scatter plots showing significant causal effects between skin microbiota and HC. **(A)** ASV013 [*S. epidermidis*]; **(B)** ASV004 [*Corynebacterium (unc.)*]; **(C)** the family: Neisseriaceae; **(D)** ASV019 [*Pasteurellaceae sp*.]; **(E)** ASV070 [*Veillonella (unc.)*]; **(F)** the phylum: Bacillota (Firmicutes); **(G)** the class: Betaproteobacteria.

Following the MR framework, we obtained a total of 595 SNPs linked with 113 bacterial genera in the KORA FF4 cohort and a total of 622 SNPs linked with 118 bacterial genera in the PopGen cohort (details provided in Supplementary Tables 1, 2). Figures 2, 3 demonstrate the correlation between skin microbiota species and PLC (Figure 2 for HBDC, Figure 3 for HC). As previously mentioned, the IVW method was chosen as the primary statistical analysis method, and after excluding genera with unknown bacterial names, a significant association was observed between nine genera and the outcome variable of PLC (Figure 4 for HBDC and Figure 5 for HC). Specifically, the family Neisseriaceae (OR = 1.13, 95% CI = 1.05–1.22, *p* = 0.0007) was found to be a risk factor for HBDC, and ASV070 [*Veillonella (unc.)*] (OR = 0.92, 95% CI = 0.87–0.99, *p* = 0.02) was found to be a protective factor for HBDC (details provided in Table 1). The class Betaproteobacteria (OR = 0.93, 95% CI = 0.86–0.99, *p* = 0.04), the phylum Bacillota (Firmicutes; OR = 0.90, 95% CI = 0.83–0.98, *p* = 0.02), and ASV070 [*Veillonella (unc.)*] (OR = 0.93, 95% CI = 0.88–0.99, *p* = 0.01) were found to be protective factors for HC. ASV013 [*S. epidermidis*] (OR = 1.16, 95% CI = 1.03–1.32, *p* = 0.02), ASV004 [*Corynebacterium (unc.)*] (OR = 1.10, 95% CI = 1.02–1.18, *p* = 0.01), the family Neisseriaceae (OR = 1.08, 95% CI = 1.01–1.18, *p* = 0.04), and ASV019 [*(Pasteurellaceae sp.)*] (OR = 1.11, 95% CI = 1.02–1.22, *p* = 0.02) showed a significant positive association with HC risk (details provided in Table 2). It is worth noting that after false discovery rate (FDR) correction, no results showed a significant association. A *p*-value above the FDR correction threshold but lower than 0.05 was considered suggestive evidence for a potential causal association. The scatter plots for the causal relationship between skin microbiota and PLC are presented in Figures 6, 7.

**Table 1:** Causal association of skin microbiota with HBDC.

**Exposure**	**Methods**	**OR**	**Low 95% CI**	**High 95% CI**	***p*-value**
*Veillonella (unc.)*	Inverse variance weighted	0.925	0.867	0.987	0.019
	Weighted median	0.941	0.864	1.025	0.161
MR-Egger	0.778	0.536	1.131	0.259
Family: Neisseriaceae	Inverse variance weighted	1.132	1.053	1.217	0.001
	Weighted median	1.126	1.020	1.243	0.018
MR-Egger	1.497	1.045	2.144	0.092

OR, odds ratio; CI, confidence interval.

**Table 2:** Causal association of skin microbiota with HC.

**Exposure**	**Methods**	**OR**	**Low 95% CI**	**High 95% CI**	***p-*value**
*S. epidermidis*	Inverse variance weighted	1.171	1.003	1.367	0.046
	Weighted median	1.165	1.028	1.320	0.017
	MR-Egger	0.710	0.092	5.480	0.798
*Corynebacterium (unc.)*	Inverse variance weighted	1.100	1.022	1.185	0.011
	Weighted median	1.086	0.987	1.195	0.090
	MR-Egger	1.381	0.909	2.097	0.269
Family: Neisseriaceae	Inverse variance weighted	1.085	1.020	1.155	0.010
	Weighted median	1.088	1.005	1.177	0.037
	MR-Egger	1.152	0.707	1.877	0.627
*Pasteurellaceae* sp.	Inverse variance weighted	1.099	1.018	1.187	0.016
	Weighted median	1.115	1.016	1.222	0.021
	MR-Egger	0.878	0.433	1.778	0.752
*Veillonella (unc.)*	Inverse variance weighted	0.933	0.883	0.986	0.014
	Weighted median	0.932	0.867	1.002	0.058
	MR-Egger	0.519	0.266	1.011	0.150
Phylum: Bacillota (Firmicutes)	Inverse variance weighted	0.905	0.833	0.983	0.019
	Weighted median	0.909	0.816	1.013	0.085
	MR-Egger	1.362	0.807	2.299	0.331
Class: Betaproteobacteria	Inverse variance weighted	0.931	0.868	1.000	0.048
	Weighted median	0.937	0.856	1.026	0.161
	MR-Egger	1.220	0.827	1.800	0.373

OR, odds ratio; CI, confidence interval.

### Sensitivity analysis

According to Cochran's *Q*-test, our IVW-MR analysis results demonstrated no evidence of heterogeneity in our findings. The MR result for ASV013 [*S. epidermidis*] on HC showed heterogeneity (*p* = 0.02). Furthermore, the MR-Egger regression analysis and MR-PRESSO analysis results provided evidence suggesting that there was no other significant horizontal pleiotropy (Tables 3, 4). We also conducted a leave-one-out analysis to identify and delete abnormal instrumental variables. The results showed the robustness of our study (Supplementary Figures 1, 2) and also suggested that the MR analysis results were relatively stable.

**Table 3:** Sensitivity analysis of skin microbiota in HBDC.

**Exposure**	***Q***	***p*-value for Cochran's *Q*-test**	**Egger-intercept**	***p*-value for MR-Egger intercept**
*Veillonella (unc.)*	5.722	0.334	0.124	0.409
Family: Neisseriaceae	1.360	0.851	−0.185	0.195

**Table 4:** Sensitivity analysis of skin microbiota in HC.

**Exposure**	***Q***	***p*-value for Cochran's *Q*-test**	**Egger-intercept**	***p*-value for MR-Egger intercept**
*S. epidermidis*	5.464	0.019	0.266	0.714
*Corynebacterium (unc.)*	0.419	0.811	−0.132	0.392
Family: Neisseriaceae	2.258	0.323	−0.030	0.831
*Pasteurellaceae* sp.	0.241	0.887	0.149	0.594
*Veillonella (unc.)*	0.956	0.812	0.384	0.182
Phylum: Bacillota (Firmicutes)	0.392	0.942	−0.182	0.219
Class: Betaproteobacteria	2.248	0.690	−0.138	0.239

## Discussion

We conducted an MR analysis to investigate the causal association between skin microbiota and PLC (HBDC and HC) using GWAS summary-level data. Our results showed that *Veillonella (unc.)* plays a protective role in HBDC, and the family Neisseriaceae has a causal risk impact on HBDC. The class Betaproteobacteria, the phylum Bacillota (Firmicutes), and *Veillonella (unc.)* play a protective role against HC. *S. epidermidis, Corynebacterium (unc.)*, the family Neisseriaceae, and *Pasteurellaceae* sp. are associated with an increased risk of HC. Our MR analysis revealed a genetic causal association between skin microbiota and PLC, indicating that the skin microbiome may take part in the progression of PLC. To the best of our knowledge, our study is the first MR analysis to investigate the potential causal association between skin microbiota and PLC.

Our MR analysis demonstrated a causal association between nine bacterial genera and PLC, while some of these bacterial genera have previously been reported to be associated with cancer. For example, *Veillonella* is reportedly associated with radiology-proven objective responses in patients with unresectable HCC (Lee et al., 2022). In addition, Zheng et al. (2023) also reported that *Veillonella* was associated with an early recurrence of hepatitis B virus (HBV)-related HC. *S. epidermidis*, which is the bacterium present on the skin, could boost tumor-specific T cells and exert cytotoxic activity (Chen et al., 2023). Additionally, Bernardo et al. (2023) also reported that *S. epidermidis* exhibits potent inflammatory activity and increases Tregs in breast cancer. Oral *Corynebacterium* is associated with a decreased risk of head and neck squamous cell carcinoma (HNSCC; Hayes et al., 2018). Furthermore, the combination of *Corynebacterium parvum* and heterologous tumor antisera reportedly extended survival longer than either modality alone in murine ovarian cancer (Knapp and Berkowitz, 1977). *Pasteurellaceae* act as salivary microbiome biomarkers in patients with oral squamous cell carcinoma (SCC; Medeiros et al., 2023).

Compared to studies on gut microbiota, there are limited studies focusing on the influence of the skin microbiota on other organs. The host genetic factors modulating the interactions between the skin and the microbiome are still largely unclear. Studies have reported the influence of the skin microbiota on skin diseases. For instance, MR results have indicated the influence of *staphylococci* in dermatitis/eczema; *Flavobacteriaceae* plays a role in microenvironment-specific effects in allergies. Additionally, *Staphylococcus* ASVs are associated with psoriasis, seborrheic keratosis, and vitiligo (Moitinho-Silva et al., 2022). In contrast, *staphylococci* showed a potential protective effect on allergic rhinitis (Moitinho-Silva et al., 2022). Skin microbiota is also associated with skin cancer (non-melanoma skin cancer, melanoma, and cutaneous T-cell lymphoma, Woo et al., 2022).

Studies have demonstrated that the microbiome plays an active role in various functions of the host, such as circadian rhythms (CRs), metabolism, and immunity, rather than simply being a passive observer. Skin microbiota is also involved in the development of the innate immune system. For example, *S. epidermidis*, a common skin commensal, has several immunity effects on the skin. It produces lipoteichoic acids that prevent inflammation caused by skin injuries (Lai et al., 2009). Additionally, *S. epidermidis* promotes the expression of antimicrobial peptides, such as human β-defensins (hBDs), and enhances the function of skin lymphocytes, thereby contributing to increased skin immunity (Naik et al., 2012; Hou et al., 2022). Zheng et al. (2020) indicated that the CD8+ T-cell response specific to *S. epidermidis* is mediated by non-classical MHC class I molecules and plays a role in tissue repair. There is growing evidence suggesting that skin damage and sensitization can have an impact on other barrier sites in the body, such as the intestines and lungs. Accordingly, when exposed to *S. aureus*, keratinocytes are stimulated to produce IL-36, which leads to an increase in serum immunoglobulin E (IgE) levels. These findings support the idea that skin exposure to microbial pathogens can trigger systemic inflammation (Harris-Tryon and Grice, 2022).

CRs are central to almost every biological process. The interconnections between CRs and metabolic syndrome contribute to the development of certain diseases (Gnocchi and Bruscalupi, 2017). Metabolic syndrome directly impacts health and increases the risk of cancer (Bishehsari et al., 2020). For instance, NAFLD is a common metabolic syndrome, as well as the most frequent chronic liver disease. Although NAFLD is considered quite benign, it can eventually progress to PLC (Gnocchi et al., 2019). Studies have indicated that CRs are involved in the regulation of hormonal and metabolic homeostases playing a role in the development and progression of NAFLD and eventually in the onset of PLC (Gnocchi et al., 2019). Recent findings have also revealed a link between CRs and microbiota, suggesting that human CRs have a deep interconnection with their microbiomes (Matenchuk et al., 2020), which potentially promotes NAFLD (Gnocchi et al., 2019; Michels et al., 2022). These findings indicate that skin microbiota may also take part in metabolic syndrome (e.g., NAFLD) and lead to PLC.

This is the first study to investigate the causal relationship between human skin microbiota and PLC using MR analysis. Our results indicated that skin microbiota could act as new biomarkers for various types of cancer. Given the easy and convenient methods for acquiring and extracting skin microbiota, our results can offer more possibilities for future cancer diagnosis and treatment.

There are several limitations to our study. First, due to the original GWAS statistics, we were unable to divide the cohorts or perform subgroup analyses. Second, our analyses only included individuals of European descent. Although using a single European population to investigate causal relationships can minimize population stratification bias, it is important to interpret these findings with caution regarding their applicability to other populations. Finally, our MR analysis reported that skin microbiota has a causal influence on PLC, but the underlying mechanism remains to be elucidated. Moreover, although our findings indicated that skin microbiota has nominal causal associations with PLC, these correlations disappeared after FDR correction was applied. However, it is important to note that FDR correction can result in false negatives (Larsson et al., 2017).

## Conclusion

In conclusion, our study has provided novel evidence that skin microbiota has a causal impact on PLC. The family Neisseriaceae is associated with an increased risk of HBDC, and *Veillonella (unc.)* is associated with a decreased risk of HBDC. The class Betaproteobacteria, the phylum Bacillota (Firmicutes), and *Veillonella (unc.)* are associated with a reduced risk of HC. *S. epidermidis, Corynebacterium (unc.)*, the family Neisseriaceae, and *Pasteurellaceae* sp. show a significant positive association with HC. These skin microbiota can serve as new biomarkers for PLC and offer novel therapeutic targets and clinical strategies for the diagnosis and treatment of PLC.

## Data availability statement

The original contributions presented in the study are included in the article/Supplementary material, further inquiries can be directed to the corresponding author/s.

## Ethics statement

Ethical approval was not required for the study involving humans in accordance with the local legislation and institutional requirements. Written informed consent to participate in this study was not required from the participants or the participants' legal guardians/next of kin in accordance with the national legislation and the institutional requirements.

## Author contributions

XW: Conceptualization, Data curation, Formal analysis, Investigation, Writing – original draft. ZZ: Data curation, Writing – original draft.

## Supplementary material

Supplementary Figure 1 The results of leave-one-out sensitivity analysis for skin microbiota in HBDC. **(A)** ASV070 [*Veillonella (unc.)*]; **(B)** the family: Neisseriaceae.

Supplementary Figure 2 The results of Leave-one-out sensitivity analysis for skin microbiota in HC. **(A)** ASV013 [*S. epidermidis*]; **(B)** ASV004 [*Corynebacterium (unc.)*]; **(C)** the family: Neisseriaceae; **(D)** ASV019 [*Pasteurellaceae sp*.]; **(E)** ASV070 [*Veillonella (unc.)*]; **(F)** the phylum: Bacillota (Firmicutes); **(G)** the class: Betaproteobacteria.
