# Supplementary figures and images for: A Mendelian randomization analysis reveals the multifaceted role of the skin microbiota in liver cancer
Source: Front Microbiol. 2024 Jul 24;15:1422132. doi: 10.3389/fmicb.2024.1422132 (PMC11303314; doi:10.3389/fmicb.2024.1422132)

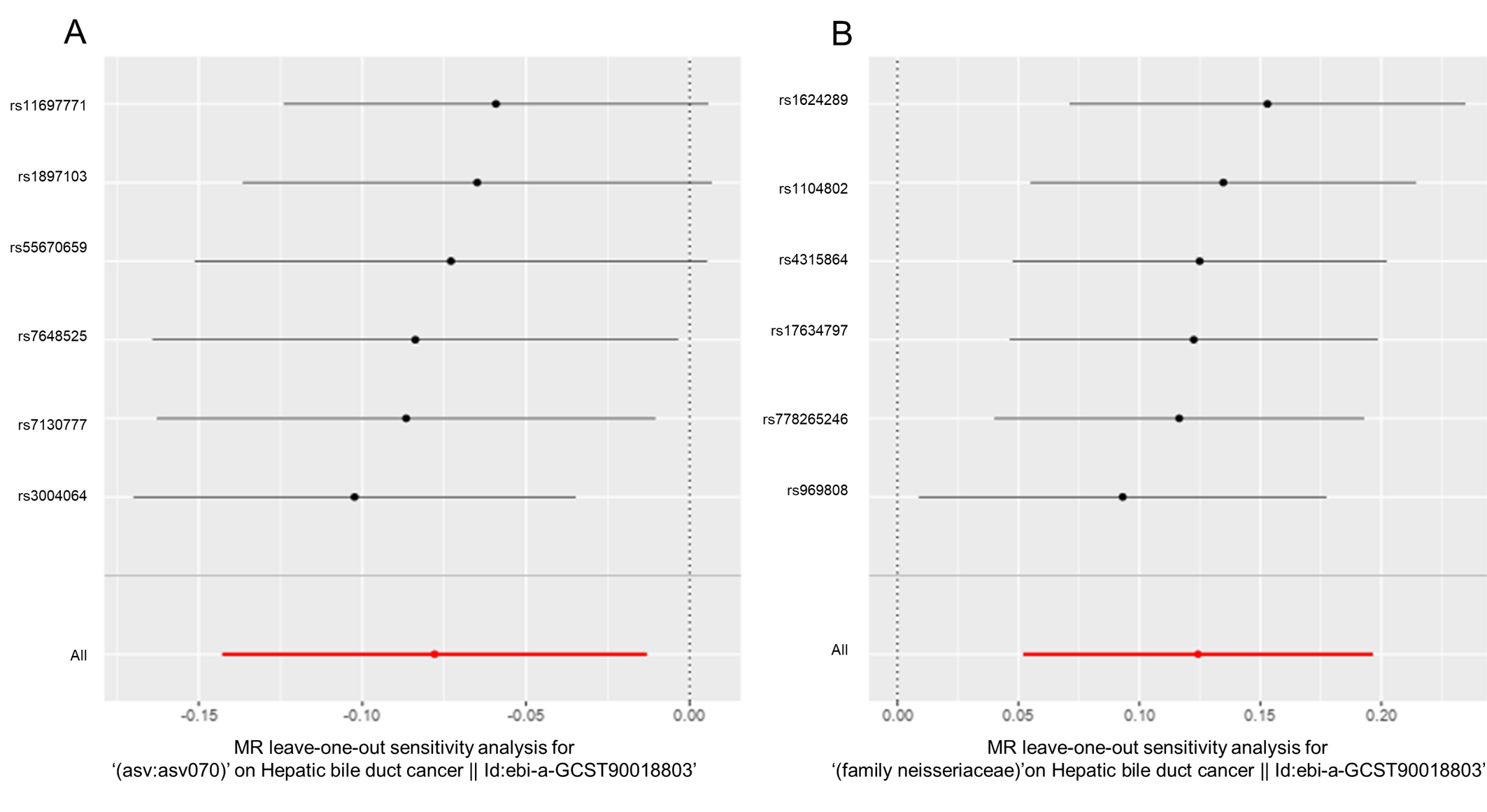

Supplement: Supplementary Figure 1 — The results of leave-one-out sensitivity analysis for skin microbiota in HBDC. (A) ASV070 [Veillonella (unc.)]; (B) the family: Neisseriaceae. [file Image_1.TIF]

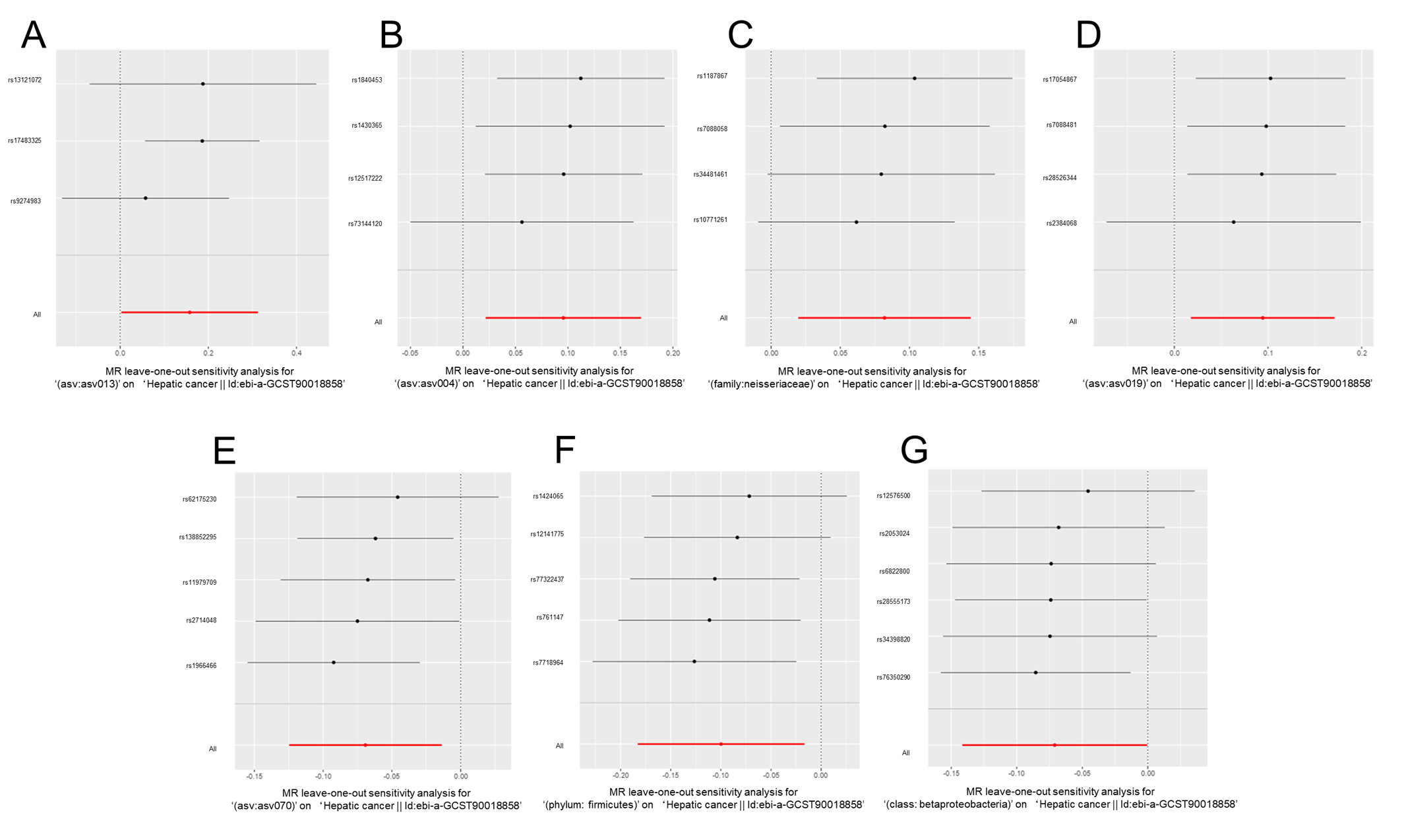

Supplement: Supplementary Figure 2 — The results of Leave-one-out sensitivity analysis for skin microbiota in HC. (A) ASV013 [S. epidermidis]; (B) ASV004 [Corynebacterium (unc.)]; (C) the family: Neisseriaceae; (D) ASV019 [Pasteurellaceae sp.]; (E) ASV070 [Veillonella (unc.)]; (F) the phylum: Bacillota (Firmicutes); (G) the class: Betaproteobacteria. [file Image_2.TIF]
